# Effectiveness and safety of oral anticoagulants for non-valvular atrial fibrillation: a population-based cohort study in primary healthcare in Catalonia

**DOI:** 10.3389/fphar.2023.1237454

**Published:** 2023-09-15

**Authors:** Maria Giner-Soriano, Dan Ouchi, Roser Vives, Carles Vilaplana-Carnerero, Andrea Molina, Antoni Vallano, Rosa Morros

**Affiliations:** ^1^ Fundació Institut Universitari per a la Recerca a l’Atenció Primària de Salut Jordi Gol i Gurina (IDIAPJGol), Barcelona, Spain; ^2^ Universitat Autònoma de Barcelona, Barcelona, Spain; ^3^ Department of Pharmacology, Therapeutics and Toxicology, Universitat Autònoma de Barcelona, Barcelona, Spain; ^4^ Medicines Department, Catalan Healthcare Service, Barcelona, Spain; ^5^ Plataforma SCReN, UIC IDIAPJGol, Barcelona, Spain; ^6^ Department of Medicine, University of Barcelona, Barcelona, Spain; ^7^ Institut Català de la Salut, Barcelona, Spain

**Keywords:** oral anticoagulants, atrial fibrillation, adherence, effectiveness, safety, electronic health records, primary healthcare, stroke

## Abstract

**Objectives:** Our objective was to analyse effectiveness and safety of oral anticoagulants (OAC) for stroke prevention in non-valvular atrial fibrillation.

**Material and methods:** Population-based cohort study including adults initiating oral anticoagulants, either direct oral anticoagulants (DOAC) or vitamin K antagonists (VKA), during 2011–2020.

**Data source:** SIDIAP, capturing information from the electronic health records of Primary Health Care in Catalonia, Spain.

**Study outcomes:** stroke, cerebral and gastrointestinal (GI) haemorrhage, assessed by patients’ subgroups according to different clinical characteristics.

**Results:** We included 90,773 patients. Male sex, older than 75, previous event, peripheral artery disease, deep vein thrombosis, or receiving antiplatelets, antidiabetics or proton pump inhibitors (PPI) was associated with higher stroke risk. For DOAC-treated, treatment switch increased stroke risk, while being adherent had a protective effect. Men, antidiabetic treatment or a previous event increased the risk of cerebral bleeding. Receiving direct oral anticoagulants had a protective effect in comparison to vitamin K antagonists. For DOAC-treated, treatment switch increased, and adherence decreased the bleeding risk. Men, people with chronic kidney disease or a previous event posed an increased risk of gastrointestinal bleeding, whereas receiving PPI had a protective effect. For DOAC-treated, switch was associated with a higher bleeding risk.

**Conclusion:** Being men, a previous event and DOAC-switch posed a higher risk for all study outcomes. direct oral anticoagulants had a protective effect against cerebral bleeding in comparison to vitamin K antagonists. Adherence to direct oral anticoagulants resulted in lower risk of stroke and cerebral bleeding. We found no differences in the risk of stroke and gastrointestinal bleeding when we compared direct oral anticoagulants vs. vitamin K antagonists.

## Introduction

Atrial fibrillation (AF) is the most common form of chronic arrhythmia. It is associated with several cardiovascular conditions, and it increases the risk of stroke. Although men are more commonly affected by AF, women have a higher risk of experiencing stroke (Lip et al., 2012; Hindricks et al., 2021). Oral anticoagulants (OAC), either vitamin K antagonists (VKA) or direct oral anticoagulants (DOAC) are usually prescribed to prevent stroke in patients with non-valvular atrial fibrillation (NVAF).

In their pivotal randomized clinical trials, all DOAC demonstrated to be at least non-inferior to warfarin in stroke prevention (Connolly et al., 2009; Granger et al., 2011; Patel et al., 2011; Giugliano et al., 2013). In recent years, multiple observational studies have analysed effectiveness and safety of DOAC in comparison to warfarin and coumarins (Anguita Sánchez et al., 2020; Durand et al., 2020; Lee et al., 2020; Crocetti et al., 2021; Lip et al., 2021; Grymonprez et al., 2023), between different DOAC (Rutherford et al., 2020; Jaksa et al., 2022; Talmor-Barkan et al., 2022), or in certain population subgroups of interest (Bang et al., 2020; Costa et al., 2020; Rodríguez-Bernal et al., 2021). Some studies have also assessed these outcomes based on the dose of DOAC or the adherence to treatment (Deshpande et al., 2018; Staerk et al., 2018; Kohsaka et al., 2020), considering that adequate levels of adherence have shown to decrease the occurrence of thromboembolic events (Amin and Marrs, 2015; Yao et al., 2016a).

We have recently analysed the baseline clinical characteristics and the sex and gender differences of patients initiating OAC for stroke prevention in NVAF from 2011–2020 in a Primary Health Care (PHC) cohort in Catalonia, Spain (Giner-Soriano et al., 2023). In the present manuscript, we have analysed the effectiveness and safety of OAC in the above-mentioned cohort, only including patients who collected their medication in the pharmacy, and assessed by different subgroups based on sex, age, renal impairment or with other frequent comorbidities and comedications; and by dose adequacy, treatment adherence or drug switch in the case of those people treated with DOAC.

## Material and methods

### Study design

**FIGURE 1:**
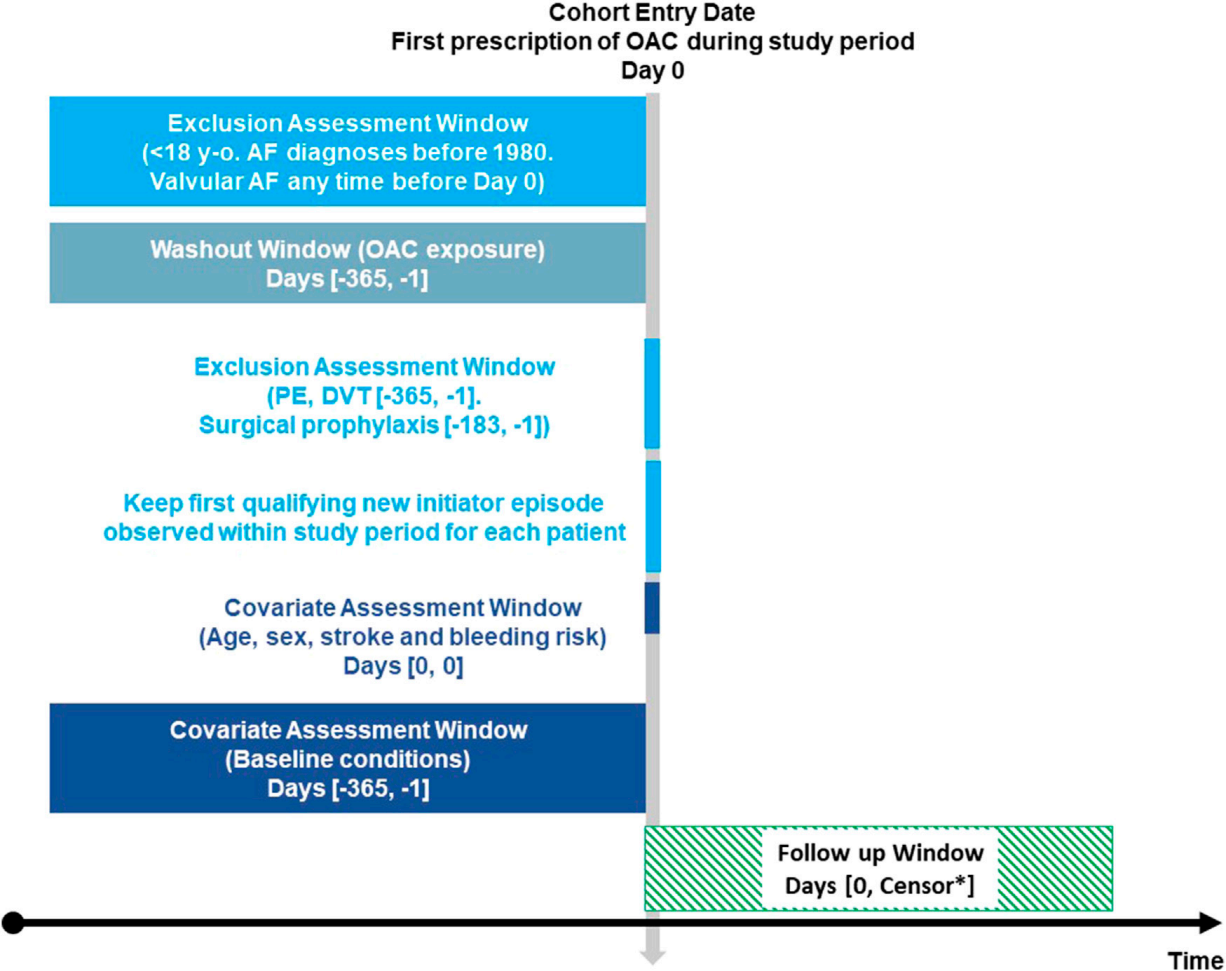
Exposure-based cohort entry Figure 1 depicts the time of variables’ assessment at cohort entry. *Earliest of: outcome of interest (stroke, cerebral or gastrointestinal bleeding), death, disenrollment, end of study period. OAC: oral anticoagulants. AF: atrial fibrillation. PE: pulmonary embolism. DVT: deep vein thrombosis. Adapted from Schneeweiss et al., 2019; Schneeweiss et al., 2019).

Population-based cohort study including adults with NVAF who initiated OAC treatment. Cohort entry criteria are explained in Figure 1.

### Population included

We included all ≥18 years-old individuals with an active diagnosis of NVAF registered in the PHC electronic records who initiated treatment with OAC from January 2011 to December 2020.

### Population excluded

We excluded from the analysis those individuals who had been diagnosed with AF before 1980, people with valvular AF, those who had experienced pulmonary embolism (PE) or deep vein thrombosis (DVT) during the previous 12 months to the OAC prescription, those receiving OAC for surgical prophylaxis of hip or knee replacement during the previous 6 months, and those with an OAC prescribed during the study period but with no subsequent dispensing during the next 120 days.

### Data source

The data source is the Information System for the Development of Research in Primary Care (SIDIAP) (Recalde et al., 2022; SIDIAP, 2022), which captures clinical information of approximately 5.8 million people from Catalonia, Spain (around 80% of the Catalan population). This information is pseudonymized, originated from different data sources:

1) ECAP (Electronic Health Records in PHC in Catalonia); including socio-demographic characteristics, residents in nursing homes/long-term care facilities, comorbidities registered as International Classification of Diseases (ICD)-10 codes (WHO, 2019), specialist referrals, clinical parameters, toxic habits, sickness leave, date of death, laboratory test data, and drug prescriptions issued in PHC, registered as Anatomical, Therapeutic, Chemical classification system (ATC) codes (WHO Collaborating Centre for Drug Statistics Methodology, 2022).
2) Pharmacy invoice data corresponding to the PHC drug prescriptions, also by ATC.
3) Database of diagnoses at hospital discharge (CMBD-HA) (Català de la Salut, 2022).

ICD-10 codes for diagnoses and ATC codes for drugs studied are included in the Supplementary file, Supplementary Tables S1 and S2, respectively.

### Drug exposure

We included all NVAF patients who initiated an OAC treatment during the study period (2011–2020) and excluded the non-initiators, who did not have any dispensing during the subsequent 120 days. The duration of pharmacy invoice records was estimated based on the number of packages dispensed, assuming each package provided coverage for 30 days, as only the month of dispensing was available.

For DOAC-treated, we assessed: dose of DOAC; defining the dose adequacy according to the Summary of Product Characteristics, SPC (Supplementary Table S3), discontinuation; defined as no dispensing during more than 2 months after initiation, persistence; defined as no discontinuation of OAC treatment, adherence to treatment; measured by Medication Possession Ratio (MPR) (Hess et al., 2006) and considering adherents those with MPR ≥80%, and treatment switch; when the first OAC was discontinued and a different one was initiated during the study period.

### Study outcomes

We estimated incidence rates (IR) of ischaemic stroke, cerebral haemorrhage, and gastrointestinal (GI) haemorrhage for all OAC initiators throughout the follow-up period. Patients were censored at the time when any of the following events occurred: outcome of interest (stroke, cerebral or GI bleeding), death, disenrollment from the database, or end of study period (Figure 1).

### Statistical analysis

In order to model the longitudinal drug exposure, we used a computational technique, the smooth algorithm. This algorithm utilizes non-parametric statistical techniques to identify the most probable treatment based on all drug dispensations documented for each patient throughout the study period (Ouchi et al., 2022).

For the effectiveness and safety analyses, we calculated IR of all outcomes of interest as the cumulative number of events per 1,000 person-year for OAC initiators. We estimated incidence rate ratios (IRR) and 95% confidence intervals (CI), crude and adjusted, by fitting a negative binomial regression for stroke, cerebral and GI bleeding. The log (time) was used as an offset in the models, and the sandwich method was employed to estimate robust standard errors. The covariables were age (≥75 years), sex, CHA_2_DS_2_VASc, previous event for each outcome of interest, comorbidities–including chronic kidney disease (CKD) defined by diagnosis and/or glomerular filtration rate –, and comedications.

We conducted subgroup analyses for those patients exposed to DOAC by dose adequacy according to the criteria in the SPC, adherence (MPR ≥80%), and treatment switch during follow-up.

All statistical analyses were conducted with R software (version 4.1 or superior) with a significance level of 5%.

## Results

**FIGURE 2:**
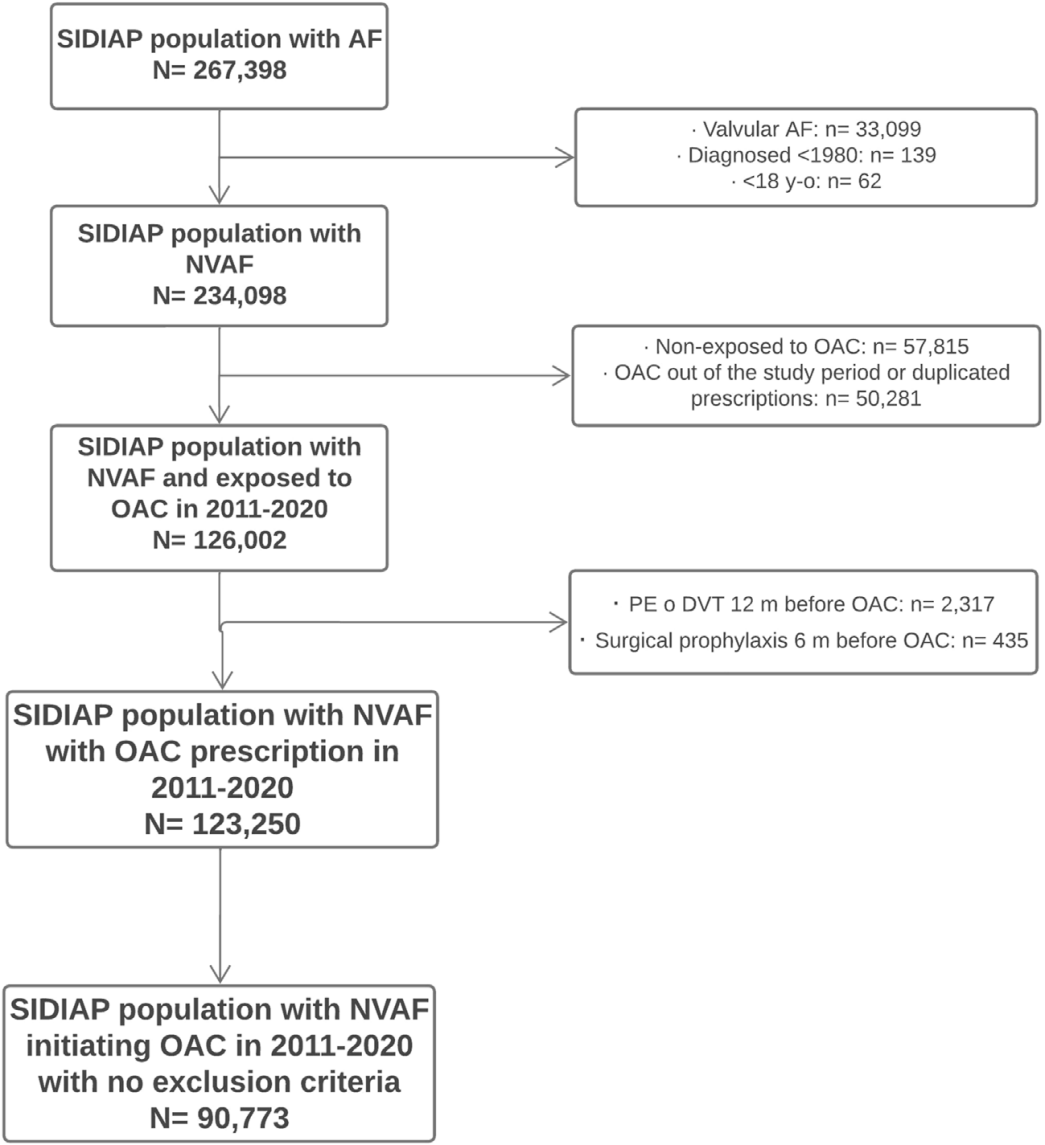
Flow diagram of population included Flowchart of patients’ inclusion in the study. SIDIAP: Information System for the Development of Research in Primary Care. AF: atrial fibrillation. NVAF: non-valvular atrial fibrillation. OAC: oral anticoagulants. PE: pulmonary embolism. DVT: deep vein thrombosis.

During the period spanning 2011 to 2020, 123,250 people with NVAF were prescribed a new OAC. Their baseline socio-demographic and clinical characteristics and the persistence and adherence to treatment have been described elsewhere (Giner-Soriano et al., 2023). Of these people, 90,773 (73.6%) received a dispensing for the OAC prescription and were included in the analyses of effectiveness and safety (Figure 2). The median follow-up time was 36.7 months (interquartile range, IQR, 17.9–61.2) and the median time to first treatment switch for all OAC was 18.7 months (IQR, 5.8–43.0).

### Effectiveness analysis

Table 1 shows the number of stroke events, IR and IRR, crude and adjusted for covariates. The overall IR of stroke was 35.9 events per 1,000 person-year (95% CI 35.1–36.7). With regards to the binomial regression of all patients treated with OAC, the factors associated with an increased risk of stroke were age older than 75 (IRR 1.33, 95% CI 1.23–1.44), male sex (IRR 1.12, 95% CI 1.05–1.20), having experienced a previous stroke–which posed the greatest risk of stroke (IRR 8.27, 95% CI 7.75–8.83) –, being diagnosed with peripheral artery disease, PAD, (IRR 1.35, 95% CI 1.22–1.49) or DVT (IRR 1.83, 95% CI 1.01–3.33), and receiving concomitant treatment with antiplatelets (IRR 1.13, 95% CI 1.05–1.22), antidiabetic drugs (IRR 1.32, 95% CI 1.23–1.42) or proton pump inhibitors, PPI, (IRR 1.13, 95% CI 1.06–1.21). There was no difference in stroke risk when comparing DOAC vs. VKA and for patients with or without CKD.

**TABLE 1:** Stroke incidence in initiators of oral anticoagulants during the study period.

		N total	N events	Sum of person-years	IR (95% CI), 1000 person/year	IRR (95% CI)	*p*-value	Adjusted IRR (95% CI)	*p*-value
**All patients (DOAC and VKA)**									
**OAC**	DOAC	36458	2814	60184	46.8 (45.0–48.5)	1.27 (1.19–1.34)	<0.001	1.02 (0.96–1.08)	0.443
	VKA	54315	4306	138385	31.1 (30.2–32.1)				
**Age**	>75	51046	4613	103500	44.6 (43.3–45.9)	1.66 (1.56–1.76)	<0.001	**1.33 (1.23–1.44)**	<0.001
	≤75	39727	2507	95069	26.4 (25.4–27.4)				
**Sex**	Men	48348	3961	103806	38.2 (37.0–39.4)	1.11 (1.05–1.18)	<0.001	**1.12 (1.05–1.20)**	0.001
	Women	42425	3159	94763	33.3 (32.2–34.5)				
**CKD**	Yes	8625	804	14731	54.6 (50.9–58.5)	1.42 (1.30–1.55)	<0.001	1.08 (0.98–1.18)	0.116
	No	75747	5964	172211	34.6 (33.8–35.5)				
**Previous event**	Yes	13818	4061	26513	153.2 (148.5–158.0)	9.30 (8.80–9.83)	<0.001	**8.27 (7.75–8.83)**	<0.001
	No	76955	3059	172056	17.8 (17,2–18,4)				
**PAD**	Yes	6293	878	11671	75.2 (70,3–80,4)	2.11 (1.93–2.31)	<0.001	**1.35 (1.22–1.49)**	<0.001
	No	84480	6242	186898	33.4 (32.6–34.2)				
**DVT**	Yes	114	17	172	98.8 (57.6–158.3)	1.99 (1.10–3.60)	0.023	**1.83 (1.01–3.33)**	0.048
	No	90659	7103	198397	35.8 (35.0–36.6)				
**Antiplatelets**	Yes	15179	1723	31318	55.0 (52.5–57.7)	1.69 (1.58–1.81)	<0.001	**1.13 (1.05–1.22)**	<0.001
	No	75594	5397	167251	32.3 (31.4–33.1)				
**Antidiabetic drugs**	Yes	22453	2446	48263	50.7 (48.7–52.7)	1.64 (1.54–1.74)	<0.001	**1.32 (1.23–1.42)**	<0.001
	No	68320	4674	150306	31.1 (30.2–32.2)				
**PPI**	Yes	48819	4575	105649	43.3 (42.1–44.6)	1.64 (1.54–1.74)	<0.001	**1.13 (1.06–1.21)**	<0.001
	No	41954	2545	92921	27.4 (26.3–28.5)				
**DOAC patients**									
**Dose initiated**	Overdosed	4975	249	6962	35.8 (31.5–40.5)	0.68 (0.57–0.80)	<0.001	0.88 (0.72–1.07)	0.187
	Underdosed	7837	685	12761	53.7 (49.7–57.9)	1.13 (1.02–1.25)	0.019	1.05 (0.94–1.17)	0.420
	Recommended dose	23646	1880	40462	46.5 (44.4–48.6)				
**MPR**	≥80%	31114	2432	56883	42.8 (41.1–44.5)	0.69 (0.62–0.78)	<0.001	**0.74 (0.65–0.83)**	<0.001
	<80%	5344	382	3301	115.7 (104.4–127.9)				
**DOAC switch**	Yes	3523	459	5836	78.7 (71.6–86.2)	1.80 (1.59–2.03)	<0.001	**2.08 (1.84–2.38)**	<0.001
	No	32935	2355	54348	43.3 (41.6–45.1)				

IR, Incidence rate per 1000 person/year. IRR, incidence rate ratio. VKA, vitamin K antagonists. OAC, oral anticoagulant treatment. CKD, chronic kidney disease, estimated by glomerular filtration rate <45 mL/min. PAD, peripheral artery disease. DVT, deep vein thrombosis. PPI, proton pump inhibitors. DOAC, direct oral anticoagulants. MPR, medication possession ratio. The bold value means statistically significant.

**FIGURE 3:**
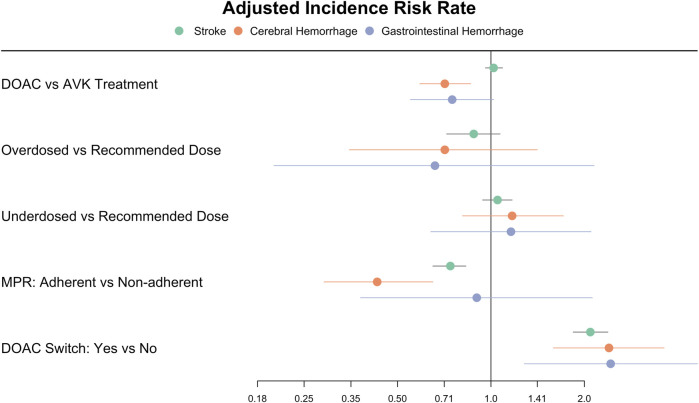
Forest plot of the incidence rate ratios of stroke, cerebral and gastrointestinal haemorrhages in patients treated with oral anticoagulants Figure 3 depicts the adjusted Incidence Rate Ratios of stroke, cerebral and gastrointestinal haemorrhages in the group of patients treated with DOAC in comparison with VKA, and in those receiving DOAC according to dose adequacy, adherence, and treatment switch. DOAC: direct oral anticoagulants. VKA: vitamin K antagonists. MPR: medication possession ratio.

For DOAC- treated patients, we found that being adherent to the treatment had a protective effect against stroke (IRR 0.74, 95% CI 0.65–0.83), whereas those who switched the DOAC during the follow-up were at increased risk (IRR 2.08, 95% CI 1.84–2.38). Receiving the correct dose of DOAC was not associated with different IR for stroke when compared under- or overdosing (Table 1; Figure 3).

### Safety analysis

#### Cerebral haemorrhage

The overall IR of cerebral haemorrhage was 3.2 events per 1,000 person-year. In reference to the results of the regression analysis (Table 2), the risk of cerebral haemorrhage increased significantly with male sex (IRR 1.49, 95% CI 1.23–1.82), previous occurrence of the event (IRR 9.26, 95% CI 6.60–12.98) and treatment with antidiabetic drugs (IRR 1.23, 95% CI 1.01–1.50), while receiving DOAC had a protective effect compared to VKA (IRR 0.71, 95% CI 0.59–0.86).

**TABLE 2:** Cerebral haemorrhage incidence in initiators of oral anticoagulants during the study period.

		N total	N events	Sum of person-years	IR (95% CI), 1000 person/year	IRR (95% CI)	*p*-value	Adjusted IRR (95% CI)	*p*-value
All patients (DOAC and VKA)									
**OAC**	DOAC	36458	185	62584	3.0 (2.6–3.4)	0.74 (0.62–0.89)	0.001	**0.71 (0.59–0.86)**	<0.001
	VKA	54315	465	142771	3.3 (3.0–3.6)				
**Age**	>75	51046	414	107582	3.9 (3.5–4.2)	1.55 (1.31–1.84)	<0.001	1.24 (1.00–1.55)	0.052
	≤75	39727	236	97773	2.4 (2.1–2.7)				
**Sex**	Men	48348	372	107381	3.5 (3.1–3.8)	1.20 (1.02–1.42)	0.029	**1.49 (1.23–1.82)**	<0.001
	Women	42425	278	97974	2.8 (2.5–3.2)				
**CKD**	Yes	8625	73	15257	4.8 (3.8–6.0)	1.42 (1.10–1.84)	0.007	1.21 (0.93–1.58)	0.148
	No	75747	551	178129	3.1 (2.8–3.4)				
**Previous event**	Yes	1153	38	2447	15.5 (11.0–21.3)	6.66 (4.69–9.46)	<0.001	**9.26 (6.60–12.98)**	<0.001
	No	89620	612	202908	3.0 (2.8–3.3)				
**PAD**	Yes	6293	48	12219	3.93 (2.9–5.2)	1.18 (0.86–1.62)	0.299	0.92 (0.66–1.27)	0.601
	No	84480	602	193136	3.1 (2.9–3.4)				
**DVT**	Yes	114	1	190	5.3 (0.1–29.3)	1.67 (0.24–11.89)	0.607	2.02 (0.28–14.45)	0.482
	No	90659	649	205165	3.2 (2.9–3.4)				
**Antiplatelets**	Yes	15179	128	32757	3.9 (3.3–4.7)	1.29 (1.05–1.59)	0.016	1.14 (0.92–1.43)	0.229
	No	75594	522	172598	3.0 (2.8–3.3)				
**Antidiabetic drugs**	Yes	22453	214	50173	4.3 (3.7–4.9)	1.56 (1.31–1.86)	<0.001	**1.23 (1.01–1.50)**	0.042
	No	68320	436	155182	2.8 (2.6–3.1)				
**PPI**	Yes	48819	386	109765	3.5 (3.2–3.9)	1.30 (1.10–1.53)	0.002	1.11 (0.94–1.33)	0.225
	No	41954	264	95590	2.8 (2.4–3.1)				
**DOAC patients**									
**Dose initiated**	Overdosed	4975	14	7126	2.0 (1.1–3.3)	0.55 (0.32–0.96)	0.036	0.71 (0.35–1.41)	0.326
	Underdosed	7837	54	13269	4.1 (3.1–5.3)	1.36 (0.97–1.92)	0.076	1.17 (0.81–1.71)	0.404
	Recommended dose	23646	127	42163	3.0 (2.5–3.6)				
**MPR**	≥80%	31114	162	59144	2.7 (2.3–3.2)	0.50 (0.33–0.74)	<0.001	**0.43 (0.29–0.65)**	<0.001
	<80%	5344	33	3415	9.7 (6.7–13.6)				
**DOAC switch**	Yes	3523	34	6221	5.5 (3.8–7.6)	1.96 (1.32–2.91)	<0.001	**2.40 (1.59–3.61)**	<0.001
	No	32935	161	56338	2.9 (2.4–3.3)				

IR, Incidence rate per 1000 person/year. IRR, incidence rate ratio. VKA, vitamin K antagonists. OAC, oral anticoagulant treatment. CKD, chronic kidney disease, estimated by glomerular filtration rate <45 mL/min. PAD, peripheral artery disease. DVT, deep vein thrombosis. PPI, proton pump inhibitors. DOAC, direct oral anticoagulants. MPR, medication possession ratio. The bold value means statistically significant.

Among patients treated with DOAC, adherence to treatment was protective against the event (IRR 0.43, 95% CI 0.29–0.65), while switching drug during follow-up resulted in an increased risk of cerebral bleeding (IRR 2.40, 95% CI 1.59–3.61), and receiving an adequate dose had no significant effect compared to under- or overdosing (Table 2; Figure 3).

#### Gastrointestinal bleeding

The overall IR of GI haemorrhage was 1.2 events per 1,000 person-year. As shown in Table 3, male sex (IRR 2.14, 95% CI 1.56–2.94), presence of CKD (IRR 1.75, 95% CI 1.17–2.62) and history of previous event (IRR 5.93, 95% CI 2.78–12.66) were associated with a higher risk of haemorrhage, while treatment with PPI (IRR 0.59, 95% CI 0.44–0.78) and a history of DVT (IRR 0, 95% CI 0–0) had a protective effect.

**TABLE 3:** Gastrointestinal haemorrhage incidence in initiators of oral anticoagulants during the study period.

		N total	N events	Sum of person-years	IR (95% CI), 1000 person/year	IRR (95% CI)	*p*-value	Adjusted IRR (95% CI)	*p*-value
All patients (DOAC and VKA)									
**OAC**	DOAC	36458	65	62644	1.0 (0.8–1.3)	0.70 (0.52–0.94)	0.018	0.75 (0.55–1.02)	0.071
	VKA	54315	180	142977	1.3 (1.1–1.5)				
**Age**	>75	51046	149	107709	1.4 (1.2–1.6)	1.34 (1.02–1.76)	0.036	1.12 (0.78–1.61)	0.543
	≤75	39727	96	97911	1.0 (0.8–1.2)				
**Sex**	Men	48348	163	107478	1.5 (1.3–1.8)	1.83 (1.38–2.43)	<0.001	**2.14 (1.56–2.94)**	<0.001
	Women	42425	82	98143	0.8 (0.7–1.0)				
**CKD**	Yes	8625	33	15256	2.2 (1.5–3.0)	1.84 (1.26–2.70)	0.002	**1.75 (1.17–2.62)**	0.007
	No	75747	202	178383	1.1 (1.0–1.3)				
**Previous event**	Yes	581	8	1143	7.0 (3.0–13.8)	6.38 (3.08–13.22)	<0.001	**5.93 (2.78–12.66)**	<0.001
	No	90192	237	204478	1.2 (1.0–1.3)				
**PAD**	Yes	6293	26	12235	2.1 (1.4–3.1)	1.87 (1.24–2.82)	0.003	1.39 (0.88–2.21)	0.158
	No	84480	219	193386	1.1 (1.0–1.3)				
**DVT**	Yes	114	0	192	0 (0–19.3)	0 (0–0)	<0.001	**0 (0–0)**	<0.001
	No	90659	245	205429	1.2 (1.1–1.4)				
**Antiplatelets**	Yes	15179	39	32822	1.2 (0.8–1.6)	1.01 (0.71–1.43)	0.961	0.89 (0.61–1.28)	0.522
	No	75594	206	172799	1.2 (1.0–1.4)				
**Antidiabetic drugs**	Yes	22453	77	50270	1.5 (1.2–1.9)	1.44 (1.08–1.92)	0.014	1.17 (0.83–1.65)	0.378
	No	68320	168	155351	1.1 (0.9–1.3)				
**PPI**	Yes	48819	108	110010	1.0 (0.8–1.2)	0.67 (0.51–0.87)	0.003	**0.59 (0.44–0.78)**	<0.001
	No	41954	137	95610	1.4 (1.2–1.7)				
**DOAC patients**									
**Dose initiated**	Overdosed	4975	4	7129	0.6 (0.2–1.4)	0.47 (0.17–1.31)	0.147	0.66 (0.20–2.15)	0.490
	Underdosed	7837	15	13304	1.1 (0.6–1.9)	1.01 (0.56–1.84)	0.961	1.16 (0.64–2.10)	0.627
	Recommended dose	23646	46	4221	1.1 (0.8–1.5)				
**MPR**	≥80%	31114	58	59230	1.0 (0.7–1.3)	0.85 (0.39–1.87)	0.690	0.90 (0.38–2.12)	0.811
	<80%	5344	7	3414	2.1 (0.8–4.2)				
**DOAC switch**	Yes	3523	14	6234	2.3 (1.2–3.8)	2.67 (1.43–4.98)	0.002	**2.43 (1.28–4.63)**	0.006
	No	32935	51	56410	0.9 (0.7–1.2)				

IR: Incidence rate per 1000 person/year. IRR: incidence rate ratio. VKA: vitamin K antagonists. OAC: oral anticoagulant treatment. CKD: chronic kidney disease, estimated by glomerular filtration rate <45 mL/min. PAD: peripheral artery disease. DVT: deep vein thrombosis. PPI: proton pump inhibitors. DOAC: direct oral anticoagulants. MPR: medication possession ratio.

For patients treated with DOAC, switching drug during follow-up was associated with an increased risk of GI bleeding (IRR 2.43, 95% CI 1.28–4.63), while correct dose compared to under- or overdosing or MPR ≥80% vs. non-adherent did not result in significantly different bleeding risk (Table 3; Figure 3).

## Discussion

In this cohort study including 90,773 people with NVAF who initiated OAC between 2011 and 2020 and with up to 10 years of follow-up, we have studied effectiveness and safety of OAC treatment, according to age categories, sex, and the presence of prevalent comorbidities and comedications. We have also investigated these outcomes for people receiving DOAC in terms of dose, adherence and treatment switch, which had not been analysed so far in our setting, although DOAC initiation already accounts for more than 50% of new treatments (Giner-Soriano et al., 2023). To obtain more accurate information on drug intake, we have conducted the analyses in the cohort of patients with OAC dispensed rather than relying solely on prescription data (Grégoire and Moisan, 2016).

The IR of stroke in our cohort was 35.9 events per 1,000 person-years, with a narrow 95% CI of 35.1–36.7. This estimate is in line with recent studies reporting IR ranging from 15.0–36.6 events per 1,000 person-years for NVAF patients treated with OAC, depending on the population characteristics and the OAC type and dose (Lee et al., 2020; Crocetti et al., 2021; Lip et al., 2021; Grymonprez et al., 2023). It should be noted that the IR of stroke may vary depending on the geographical area and the healthcare system in which the study is conducted. In addition, differences in IR across studies may reflect variations in patient characteristics, comorbidities, and healthcare practices, as well as differences in the accuracy and definition of stroke events. Therefore, caution should be exercised when comparing IR between studies in different populations or settings.

Even so, our study provides valuable information on stroke IR in NVAF patients treated with OAC in Catalonia and highlights the importance of optimising OAC therapy to prevent these serious complications. Our results on effectiveness showed no differences in stroke risk when all DOAC-treated were compared with VKA-treated, as also found by Anguita-Sánchez et al. (Anguita Sánchez et al., 2020) and Sjögren et al. (Sjögren et al., 2017) but different to other authors who found DOAC to be protective against stroke compared to VKA (Durand et al., 2020; Lee et al., 2020). Several authors have found protection against stroke with DOAC vs. VKA when analysed by active substance (Deitelzweig et al., 2017; Halvorsen et al., 2017; Hernandez et al., 2017; Bang et al., 2020; Kohsaka et al., 2020; Crocetti et al., 2021; Lip et al., 2021; Grymonprez et al., 2023).

As mentioned above, these results must be interpreted with caution, as they might be influenced by several factors not related with drugs, such as the healthcare system, and other variables must be considered, such as adherence, doses or INR values. We found that older than 75, male, or those who had experienced a prior stroke had a higher risk of stroke. Regarding these patients with a prior history of stroke, it is necessary to highlight the critical role of this factor in predicting future stroke risk. Other authors have described stroke rates based on similar categories and found heterogeneous results (Bengtson et al., 2017; Rodríguez-Bernal et al., 2021; Jaksa et al., 2022). For DOAC-treated, adherence showed a protective effect against stroke, in line with similar studies with DOAC (Yao et al., 2016a; Deshpande et al., 2018).

Regarding cerebral bleeding, DOAC were protective compared to VKA, in line with most studies showing DOAC as the safest option with respect to cerebral haemorrhage risk (Halvorsen et al., 2017; Forslund et al., 2018; Bang et al., 2020; Durand et al., 2020; Lee et al., 2020; Rodríguez-Bernal et al., 2021; Grymonprez et al., 2023). We found an increased risk of cerebral haemorrhage for males, people with a previous event or people receiving antidiabetic drugs, being these results also heterogeneous with respect to the studies mentioned above (Bengtson et al., 2017; Rodríguez-Bernal et al., 2021; Jaksa et al., 2022). As in the case of stroke, those who had previously experienced a cerebral haemorrhage had a significantly higher risk of experiencing a new event compared to those without this antecedent. Again, optimal adherence to DOAC showed a protective effect against this outcome. Other studies analysing the impact of adherence on DOAC safety did not demonstrate a protective effect against major bleeding (Deshpande et al., 2018) or intracranial haemorrhage (Yao et al., 2016a).

With regards to GI bleeding, we found no significant differences between DOAC and VKA, in line with other studies (Bengtson et al., 2017; Durand et al., 2020; Rodríguez-Bernal et al., 2021), although multiple studies showed a favourable profile for DOAC in general, especially for apixaban, compared to other OAC (Abraham et al., 2017; Deitelzweig et al., 2017; Hernandez et al., 2017; Staerk et al., 2018; Vinogradova et al., 2018; Bang et al., 2020; Rutherford et al., 2020; van Ganse et al., 2020; Grymonprez et al., 2023). In our study, males and CKD patients or with a previous haemorrhage were at increased risk of this event, as in other studies (Keskar et al., 2017; Kumar et al., 2018), and receiving PPI had a protective effect against this outcome, as has been widely demonstrated (Ahn et al., 2022). DOAC adherence had no protective effect against GI bleeding compared to non-adherence. Yao et al. analysed effectiveness and safety according to treatment persistence and found that, for those with CHA_2_DS_2_-VASc ≥2, being persistent for at least 6 months had a protective effect in front of GI bleeding. They also analysed adherence, but not its effects on the outcomes (Yao et al., 2016a).

For DOAC-treated, switching increased the risk of all three outcomes of study. Unfortunately, we were not able to ascertain the reasons for the switch, which might be contributing to these higher risks of events. It is usual in similar studies to exclude or to censor patients who switch treatment during follow-up (Crocetti et al., 2021; Grymonprez et al., 2023).

We could not find a protective effect against any of the events when an adequate dose of DOAC was prescribed in comparison to an inadequate one. As we had previously described non-despicable numbers of under- and overdosed patients according to the SPC (Giner-Soriano et al., 2023), we aimed to analyse if those people treated with inadequate doses might have associated worst clinical outcomes. Thus, we had previously hypothesized that those individuals receiving a lower dose than recommended could have shown an increased risk of stroke, but we did not find significant differences when they were compared to those receiving an adequate dose of DOAC. For haemorrhages, we could have expected higher risk for overdosed patients and lower risk for underdosed, but once more, the differences were not statistically significant. Nevertheless, we only analysed the first dose prescribed but not further changes in posology. Other authors have analysed the effects of the initial dose on the effectiveness and safety, splitting by standard and reduced dose for each DOAC, but none of them have evaluated these effects by the dose adequacy (Li et al., 2017; Nielsen et al., 2017; Staerk et al., 2018; Vinogradova et al., 2018; Kohsaka et al., 2020).

## Strengths and limitations

Strengths of our study include the long follow-up and the number of patients included from a database which has already demonstrated to be representative of the Catalan population (Recalde et al., 2022). We have analysed all four approved DOAC, in new and old users of OAC, have analysed the impact of treatment adherence or switch on the outcomes, and studied all the doses authorised in NVAF and their adequacy according to the SPC, which had not been evaluated in other studies (Li et al., 2017; Nielsen et al., 2017; Staerk et al., 2018; Vinogradova et al., 2018; Kohsaka et al., 2020).

The study also places some limitations due to the observational nature of the data, such as the potential unexamined confounding variables, missing values, or coding errors, which might have introduced bias into the study, but which are present in all database observational studies. The most important limitation is the under-registration of GI haemorrhages in CMBD database, as it captures diagnoses at hospital discharge, but in our setting most GI haemorrhages are attended and treated in short-stay hospital wards of the Emergency Departments which do not routinely register those diagnoses in the CMBD database, as they use their own database, to which we did not have access to. In fact, IR of cerebral haemorrhage have been widely documented to be lower than IR of GI haemorrhage, and this was not the case in our study (see Tables 2; 3) (Yao et al., 2016b; Halvorsen et al., 2017; Durand et al., 2020; Rodríguez-Bernal et al., 2021; Grymonprez et al., 2023). Another limitation is that we have not analysed the mortality due to the inability of capturing the cause of death in our database.

Our results should be considered hypothesis-generating due to their observational design but they give us insight into how OAC are used in clinical practice and may help to design interventions to improve dose adequacy or adherence to treatment.

Further analyses may include the study of VKA discontinuations and adherence and the impact of time in therapeutic range on the clinical outcomes or include proxies of the cause of death to study the mortality rates in the OAC-treated population.

## Conclusion

Our study in a cohort of patients with NVAF treated with OAC revealed that those with a history of previous events (stroke, cerebral and GI haemorrhage) and male patients had a higher risk for all study outcomes. For DOAC-treated, switching DOAC during follow-up was associated with an increased risk of all outcomes.

We observed a protective effect of DOAC against cerebral haemorrhage when compared to VKA. Adherence to DOAC treatment resulted in lower risks of both stroke and cerebral haemorrhage.

When compared DOAC and VKA, we did not find any substantial differences in the risk of stroke and GI bleeding.

These findings highlight the importance of considering patients’ baseline characteristics and comorbidities when prescribing OAC. Clinicians should exercise caution when prescribing OAC to patients with a history of stroke, cerebral or GI haemorrhage, older patients, men, and those with PAD or DVT, as they are at an increased risk of adverse events. Adherence to DOAC treatment and avoiding switching DOAC during follow-up could help to reduce the risk of stroke and cerebral haemorrhage.

## Data Availability

The raw data supporting the conclusion of this article will be made available by the authors, without undue reservation.

## Abbreviations

AF, atrial fibrillation; ATC, Anatomical, Therapeutic, Chemical classification system; CI, confidence interval; CKD, chronic kidney disease; CMBD, minimum dataset of diagnoses at hospital discharge; DOAC, direct oral anticoagulants; DVT, deep vein thrombosis; ECAP, electronic health records in Primary Care in Catalonia; GI, gastrointestinal; ICD-10, International Classification of Diseases 10th version; IR, incidence rate; IRR, incidence rate ratio; MPR, medication possession ratio; NVAF, non-valvular atrial fibrillation; PAD, peripheral artery disease; PE, pulmonary embolism; PHC, Primary Health Care; PPI, proton pump inhibitors; SIDIAP, Information System for the Development of Research in Primary Care; VKA, vitamin K antagonists.
