# Supplementary material for: Effectiveness and safety of oral anticoagulants for non-valvular atrial fibrillation: a population-based cohort study in primary healthcare in Catalonia
Source: Front Pharmacol. 2023 Sep 15;14:1237454. doi: 10.3389/fphar.2023.1237454 (PMC10540223; doi:10.3389/fphar.2023.1237454)
Supplement: Supplementary file 1 [file DataSheet1.docx]

# Supplementary file

## Supplementary table 1. ICD-10 codes for diagnoses and comorbidities

| **DIAGNOSES** | **ICD-10 CODES** |
| --- | --- |
| **INCLUSION CRITERIA** | |
| Atrial fibrillation | I48 |
| **EXCLUSION CRITERIA** | |
| Deep vein thrombosis | I82 |
| Pulmonary embolism | I26 |
| Valvular disease | I05, I08 |
| Surgical prophylaxis, hip replacement | 0S2, 0SB9, 0SBB, 0SC9, 0SCB, 0SH9, 0SHB, 0SQ9, 0SQB, 0SR9, 0SRA, 0SRB, 0SRE, 0SRR, 0SRS, 0SS9, 0SSB, 0ST9, 0STB, 0SW9, 0SWA, 0SWB, 0SWE, 0SWR, 0SWS |
| Surgical prophylaxis, knee replacement | 0S2, 0SBC, 0SBD, 0SCC, 0SCD, 0SHC, 0SHD, 0SQC, 0SQD, 0SRC, 0SRD, 0SRT, 0SRU, 0SRV, 0SRW, 0SSC, 0SSD, 0STC, 0STD, 0SWC, 0SWD, 0SWT, 0SWU, 0SWV |
| **COMORBIDITIES** | |
| Cancer | C00-C97 |
| Chronic kidney disease | N18 |
| Diabetes mellitus | E10-E14 |
| Dislypidaemia | E78 |
| Gastrointestinal haemorrhage | K25.0, K25.2, K25.4, K25.6, K26.0, K26.2, K26.4, K26.6, K27.0, K27.2, K27.4, K27.6, K28.0, K28.2, K28.4, K28.6, K29.0, K29.6, K29.7 |
| Heart failure | I50 |
| Hypertension | I10-I15 |
| Intracranial haemorrhage | I61-I62 |
| Ischaemic heart disease | I20-I25 |
| Liver disease | K70-K77 |
| Peripheral artery disease | I70, I73, I74 |
| Stroke | I63-I69 |
| Subaracnoidal haemorrhage | I60 |
| Transient ischaemic attack | G45 |

## Supplementary table 2. ATC codes for drugs of interest and comedications

| **DRUGS OF STUDY** | |
| --- | --- |
| Acenocoumarol | B01AA07 |
| Apixaban | B01AF02 |
| Dabigatran | B01AE07 |
| Edoxaban | B01AF03 |
| Rivaroxaban | B01AF01 |
| Warfarin | B01AA03 |
| **COMEDICATIONS** | |
| Angiotensin converting enzyme inhibitors | C09A, C09B |
| Angiotensin II receptor blockers | C09C, C09D |
| Antiarrhythmics and digoxin | C01A, C01B |
| Antihypertensive drugs | C02 |
| Antiplatelets | B01AC |
| Beta blockers | C07 |
| Calcium antagonists | C08CA, C08D |
| Diuretics | C03 |
| Drugs for diabetes | A10 |
| Nitrates | C01DA |
| Non-steroidal anti-inflammatory drugs | M01A, N02BA, N02BB |
| Other anticoagulants | B01AB, B01AD, B01AX |
| Proton pump inhibitors | A02BC |
| Statins and lipid modifying agents | C10 |
| Systemic corticosteroids | H02 |

## Supplementary table 3. Conditions for dose reduction of direct oral anticoagulants according to the Summary of Product Characteristics

| **Drug** | **Full dose recommended** | **Dose reduction conditions** |
| --- | --- | --- |
| **Apixaban** | **5 mg/12h** | **2.5 mg/12h** when at least one of the following criteria is met at the treatment start:  - 2 of 3: i) serum creatinine ≥ 1.5 mg/dL; ii) age ≥ 80; iii) weight ≤ 60 kg  - Glomerular filtration rate (GFR) < 30 mL/min (not recommended if < 15 mL/min) |
| **Dabigatran**  *75 mg dose is not authorized for non-valvular atrial fibrillation (NVAF) | **150 mg/12h** | **110 mg/12h** when any of the following criteria is met at the treatment start:  - Age ≥ 80  - GFR = 30-50 mL/min (contraindicated if < 30 mL/min)  - Simultaneous treatment with verapamil |
| **Edoxaban**  *15 mg dose is not authorized for NVAF | **60 mg/24h** | **30 mg/24h** when any of the following criteria is met at the treatment start:  - GFR = 15-50 mL/min (contraindicated if < 15 mL/min)  - Weight ≤ 60 kg  - Treatment with glycoprotein P inhibitors (cyclosporine, dronedarone, erythromycin) |
| **Rivaroxaban**  *10 mg dose is not authorized for NVAF | **20 mg/24h** | **15 mg/24h** if GFR < 50 mL/min at the treatment start (contraindicated in < 15 mL/min). |
